# Use of the English Health Literacy Questionnaire (HLQ) with Health Science University Students in Nepal: A Validity Testing Study

**DOI:** 10.3390/ijerph19063241

**Published:** 2022-03-09

**Authors:** Shyam Sundar Budhathoki, Melanie Hawkins, Gerald Elsworth, Michael T. Fahey, Jeevan Thapa, Sandeepa Karki, Lila Bahadur Basnet, Paras K. Pokharel, Richard H. Osborne

**Affiliations:** 1 Department of Primary Care and Public Health, School of Public Health, Imperial College London, St. Mary’s Campus, London W2 1PG, UK; 2 Nepalese Society of Community Medicine, Lalitpur 44700, Nepal; 3 Centre for Global Health and Equity, School of Health Sciences, Swinburne University of Technology, Melbourne, VIC 3122, Australia; melaniehawkins@swin.edu.au (M.H.); gelsworth@swin.edu.au (G.E.); rosborne@swin.edu.au (R.H.O.); 4 Department of Health Sciences and Biostatistics, School of Health Sciences, Swinburne University of Technology, Melbourne, VIC 3122, Australia; mt_fahey@hotmail.com; 5 Department of Biostatistics and Clinical Trials, Peter Macallum Cancer Centre, Melbourne, VIC 3000, Australia; 6 Department of Community Health Sciences, Patan Academy of Health Sciences, Lalitpur 44700, Nepal; linktojeevan@gmail.com; 7 Department of Health Services, Epidemiology and Disease Control Division, Ministry of Health and Population, Kathmandu 44600, Nepal; sandeepakarki07@gmail.com; 8 Department of Health Services, Curative Service Division, Ministry of Health and Population, Kathmandu 44600, Nepal; drlbbasnet@gmail.com; 9 School of Public Health and Community Medicine, B.P. Koirala Institute of Health Sciences, Dharan 56700, Nepal; paras.k.pokharel@gmail.com

**Keywords:** health literacy development, health literacy questionnaire (HLQ), health literacy measurement, non-native English users, Nepal, Standards for Educational and Psychological Testing, university students, validation study

## Abstract

Research evidence shows that health literacy development is a key factor influencing non-communicable diseases care and patient outcomes. Healthcare professionals with strong health literacy skills are essential for providing quality care. We aimed to report the validation testing of the Health Literacy Questionnaire (HLQ) among health professional students in Nepal. A cross-sectional study was conducted with 419 health sciences students using the HLQ in Nepal. Validation testing and reporting were conducted using five sources outlined by ‘the 2014 Standards for Educational and Psychological Testing’. The average difficulty was lowest (17.4%) for Scale *4*. *Social support for health*, and highest (51.9%) for Scale *6*. *Ability to actively engage with healthcare providers*. One factor Confirmatory Factor Analysis (CFA) model showed a good fit for Scale *2*, Scale *7* and Scale *9* and a reasonable fit for Scale *3* and Scale *4*. The restricted nine-factor CFA model showed a satisfactory level of fit. The use of HLQ is seen to be meaningful in Nepal and warrants translation into native Nepali and other dominant local languages with careful consideration of cultural appropriateness using cognitive interviews.

## 1. Introduction

Health literacy is a multidimensional concept that encompasses an individual’s, a family’s or a community’s knowledge, confidence and comfort (which accumulate through daily activities, social interactions and across generations) to access, understand, appraise, remember and use information about health and healthcare [1]. Heath literacy responsiveness describes the way in which policies, services, environments and providers make health information and healthcare available and accessible to people with different health literacy strengths, needs and preferences [2]. Environments, including clinical environments, enable health literacy development to increase the knowledge, confidence and comfort of individuals and communities to manage their health and make a healthy choice the easy choice [1]. In the past 20 years, health literacy research has been largely correlational, where causal links are difficult to determine between health literacy and specific health behaviours or health outcomes [3]. However, recent longitudinal and intervention research is providing increasing evidence that health literacy is a determinant of health outcomes [4,5,6,7,8]. These studies are predominantly from upper-middle- and high-income countries. Hence, there is a dearth of evidence from low-income countries about health literacy and health outcomes. In countries where health systems are under-resourced, an in-depth and nuanced understanding of the local health literacy strengths, needs and preferences of communities using a multidimensional health literacy questionnaire could be an effective way of matching needs to resources to maximize the utilization of available healthcare resources for improving population health outcomes. In an age of technology and swift exchange of information via digital platforms, it can be challenging for people to manage the quantity of information (and misinformation) and understand and use the information for immediate decision making and future wellbeing. The health literacy responsiveness of healthcare services and providers to local needs is of the utmost importance to facilitate people’s access to and understanding of health information and to support their capacity to use information and services effectively to make appropriate health decisions [2,9,10,11].

Understanding the health literacy strengths, needs and preferences of individuals, communities and populations has proven useful to support healthcare providers to respond to the needs of the people they serve [12,13,14]. Recently, research has expanded to include measurement of the health literacy of healthcare professionals and of students of the healthcare professions in efforts to increase their awareness and knowledge of health literacy [15,16,17]. If healthcare professionals themselves have health literacy needs, then this can hinder their abilities to support their patients. In an era that has a strong focus on patient-centred care, it becomes critical that healthcare professionals understand and can detect, accommodate and respond appropriately to the health literacy diversity of their communities [18,19,20]. The measurement of the health literacy strengths, needs and preferences of students studying the health professions enables early support for students’ own health literacy and helps to identify and address gaps in the curriculum for their health literacy training for their future roles as healthcare professionals [15,21]. There is a growing consensus that health sciences education curricula should not only focus on producing graduates that are able to identify people with low health literacy but also equip them with skills to engage with their patients in a fair and equitable manner [22]. Interprofessional education in health sciences is considered a potential mechanism for improving the health literacy of students [23].

The World Health Organization (WHO) Shanghai Declaration holds health literacy as one of the three pillars of health promotion to achieve sustainable health development by 2030 [24], and several countries have now incorporated health literacy into national health policy [25]. A challenge for health literacy-related policy is the generation of health literacy data that provide clear evidence on what stakeholders need to do in order to enact and deliver on health policies that improve health. Evidence is generated from specific instruments to measure health literacy and these range from tests of functional health literacy (reading, numeracy and comprehension) developed for clinical purposes [26,27,28] to self-report instruments that measure the multidimensional nature of the construct of health literacy [29,30].

Research about the properties of instruments is important to help researchers and policymakers understand which instruments are useful for intended decision-making purposes (e.g., community health initiatives, and national policy). Assumptions underlying the use of a measurement instrument in contexts that are different from that in which it was developed include that the instrument will measure the same construct in the same way across contexts, despite cultural, linguistic or other differences [31].

Nepal is classified amongst the least developed countries by the United Nations. The life expectancy at birth in Nepal is 68 years. The country’s population is 26.4 million, with 83% living in rural areas. One-fourth of the population lives below the poverty line and the adult literacy rate is 66%; however, the literacy rate in females is lower, at 57%. The doctor to population ratio in Nepal is 0.37/1000 people (as low as 0.008 in rural areas and 1.5/1000 people in the capital city). Individuals bear 55% of total healthcare expenditure as out-of-pocket payments [32]. About two-thirds of healthcare in the acute sector is provided by private hospitals. While Nepal still faces a burden of infectious diseases, it is struggling with inadequate basic hygiene and sanitation, the burden of non-communicable diseases (NCDs) is also on the rise. Health science students will increasingly find themselves needing to provide and communicate information to patients about health conditions that show no visible signs or symptoms until well established. Challenges associated with the prevention and control of NCDs can be numerous and health literacy development is now a recognized factor influencing NCD outcomes [1].

There is little research about health literacy in Nepal and there is a dearth of literature about the health literacy needs of the people of Nepal [33]. Even though Nepali is the official spoken language for all 125 ethnic groups in Nepal, there are 123 spoken languages registered in Nepal [32]. Health sciences education is conducted completely in English, which is the academic language used in all universities in Nepal. These students are likely to work in both community health and clinical settings after graduating from their university studies. The professional working language amongst doctors in Nepal is also English.

### Rationale

The Health Literacy Questionnaire (HLQ) was developed by Osborne et al. in Australia using a validity-driven approach [30,34]. The nine domains of the HLQ are designed to measure the experiences people have when they engage in understanding, accessing and using health information and health services. In its development context, the HLQ was found to have strong construct validity, reliability and acceptability to clients and clinicians [30,35] and confirmatory factor analysis (CFA) has found that the items and scales in HLQ translations tend to behave in comparable ways to the English-language HLQ [36,37,38,39], even among disparate groups in low-resource settings [40,41,42]. HLQ data are used in pre-post evaluations and to develop profiles of health literacy strengths and needs to describe the health literacy of individuals and whole populations [12,43]. These health literacy profiles can be used within the Ophelia (Optimising Health Literacy and Access) process to inform community co-design initiatives to build interventions that are fit for purpose and appropriate to specific health literacy needs in specific contexts [4,12,15,30,37,44,45,46,47,48]. Although the HLQ has been translated to Nepali, this study used the original English HLQ for assessing the health literacy of the health science students [30] because English is the official language of instruction in health sciences education and for communication between professionals in universities in Nepal.

This paper reports validity evidence from a secondary analysis of data collected from Nepalese university health science students [15]. This paper aims to evaluate the validity of the data from the English HLQ for the purpose of assessing the health literacy strengths and needs of a population of health science students in a university in Nepal.

## 2. Materials and Methods

### 2.1. The Study Setting and the Population

This study was conducted on university students enrolled for undergraduate and postgraduate studies at the largest health sciences university in Nepal. The university offers education through a community-based curriculum and students enrolled are from Nepal and India. All students enrolled at the university with undergraduate and postgraduate health sciences degrees were eligible for recruitment in the study. Any students taking courses/training that did not lead to a university degree were excluded.

### 2.2. The Health Literacy Questionnaire (HLQ)

The HLQ is a tool with strong psychometric properties [30] that was developed in Australia through consultation with community members, health practitioners and policymakers. The HLQ is widely used around the world and has undergone psychometric evaluation in a variety of contexts, including in university student settings [49].

There are 44 items across nine scales in the HLQ with each scale measuring a distinct element of health literacy construct, both conceptually and psychometrically [30]. The scales are:

1. Feeling understood and supported by healthcare providers (4 items);
2. Having sufficient information to manage my health (4 items);
3. Actively managing my health (5 items);
4. Social support for health (5 items);
5. Appraisal of health information (5 items);
6. Ability to actively engage with healthcare providers (5 items);
7. Navigating the healthcare system (6 items);
8. Ability to find good health information (5 items);
9. Understanding health information well enough to know what to do (5 items).

The HLQ measures the scores in a 4-point Likert-type response (Strongly disagree, Disagree, Agree, and Strongly agree) for the first five scales, later referred to as Part 1 scales. There is a 5-point response for the latter four scales, where the items ask about the difficulty in undertaking a task (ranging from Cannot do to Very easy), later referred to as Part 2 scales. Measurement with the HLQ results in 9 scale scores that form profiles of health literacy strengths and needs.

In this study, HLQ data will be interpreted according to the item intents of the 44 HLQ items and the high and low descriptors of the nine HLQ domains [30,47]. The HLQ scores will be used to create profiles of health literacy strengths and needs of the health science students to inform the development of health literacy initiatives.

### 2.3. Theoretical Framework

Questionnaire adaptation and translation methods usually include validity testing methods to confirm or refute conceptual and measurement equivalence across languages and cultures [47]. Validation is defined by the 2014 Standards for Educational and Psychological Testing (the Standards) as the *process* of ‘…accumulating relevant evidence to provide a sound scientific basis for the proposed score interpretations’ [50]. Validity is the extent to which evidence and the theory of the construct being measured support the proposed interpretation and use of an instrument’s scores [51,52]. Validity testing is akin to hypothesis testing: first, state the proposed interpretation and use of scores (an interpretive argument); then, evaluate existing evidence or collect new evidence to determine the extent to which the evidence supports the interpretive argument [47,53]. The Standards describes the need for evidence-based on five sources: test content; the response processes of respondents and users; the internal structure of the measurement instrument; relations to other variables; and the consequences of testing (as related to a source of invalidities such as construct under-representation or construct-irrelevant variance) [51]. These five sources of evidence rely on qualitative and quantitative research methods to generate evidence to establish if and how respondents understand and engage with the content and concepts of an instrument; the extent to which item inter-relationships conform to the intended construct; the patterns of relationships of scores to other variables as predicted by the intended construct; and the extent to which the intended consequences of testing are realized (and if there are unintended consequences of testing, as related to validity) [47,51,52]. It is argued that the use of contemporary validity testing frameworks improves the transparency of the evaluation of validity evidence, which supports other potential users of the measurement instrument [31].

### 2.4. Sampling and Data Collection

A total of 700 students who were contactable through email and Facebook messages were invited to the study between February and July 2015. Details on sampling and recruitment can be found in a previously published paper [15]. All participants provided their informed consent before they participated in the study. Ethical approval for this research was obtained from the Human Participants Ethics Committee (2013/010790) of the University of Auckland and the Institutional Review Committee (IRC430/014) at the B. P. Koirala Institute of Health Sciences. A licence to administer the HLQ was obtained from Deakin University, Australia.

This validation paper is a part of a published research study that explains the data collection process in detail [15]. The decision to use an online version of HLQ was supported by a study at the same university that showed that 92% of the students accessed the internet every day [54].

Cognitive interviews (pre-testing) were conducted using the English HLQ among 10 university students and 5 researchers (including student educators) in order to explore if the English HLQ required a linguistic or cultural adaptation. Information about the comprehensibility of the questions, students’ understanding of questions and their reasons for selecting their responses was collected. The interviews were recorded after taking informed consent.

### 2.5. Data Analysis

Descriptive statistics and plots were used to examine the demographic characteristics of the sample and, for each item, to examine the extent of missing data, the presence of floor or ceiling effects, and the HLQ item distributions.

Initially, nine separate single-factor CFA models based on the items hypothesized to measure each of the HLQ scales were fitted to the data without any additional residual correlations (RCs) among the items. Following this, a highly restricted nine-factor CFA model with neither residual correlations (RCs) among items nor cross-loading (XLs) of items across scales was fitted to the data. Modification indices associated with models were used to improve the model fit by adding RCs or XLs according to the following strategy:

1. Identify a single RC to add for each scale based on the greatest standardized expected parameter change (SEPC) associated with each of the single-factor CFA models in the case that the model did not fit according to the overall chi-square test.
2. Fit modified nine-factor CFA model, allowing correlations among factors and with both (a) the RCs identified in step 1 added and (b) the single XLs from the restricted nine-factor CFA model having largest SEPC for items 32 and 36.
3. Identify a single XL to add based on the greatest SEPC reported for the CFA model in step 2 and refit a new unrestricted nine-factor CFA model including this XL.
4. Repeat step 3 adding a single XL iteratively to the new modified CFA model until model fit is achieved (CFI ≥ 0.95 and RMSEA ≤ 0.05).
5. Remove any standardized target loadings (TLs), XLs or RCs that are close to zero (TLs ≤ 0.10, RCs ≤ 0.05 in absolute value) from the final model.

All CFA models were fitted using the weighted least squares mean and variance adjusted (WLSMV) estimator for ordinal data available in Mplus version 8.4. Goodness of fit was reported using the overall model chi-square test, the comparative fit index (CFI), the Tucker–Lewis index (TLI), and the root mean square error of approximation (RMSEA).

#### 2.5.1. Test Content

Evidence for the development of the content of the HLQ items has been thoroughly investigated in previous studies [30,55]. In this study, item difficulty was investigated as an indicator of the extent to which respondents understood the content of each item. For each item of the HLQ, the difficulty level was measured, which describes how easy or difficult it is for the respondents to score high. The difficulty level for Part 1 of the HLQ (Scales *1*–*5*) was calculated as the fraction of ‘*disagree*/*strongly disagree*’ responses as against ‘*agree*/*strongly agree*’. The difficulty level for Part 2 of the questionnaire (Scales *6*–*9*) was calculated as the fraction responding ‘*cannot do or always difficult/usually difficult*/*sometimes difficult*’ against ‘*usually easy/always easy*’.

#### 2.5.2. The Response Processes of Respondents and Users

Cognitive interviews with students and staff which examined the extent to which respondents engaged with the concepts and meanings of the English-language items (i.e., evidence-based on response processes), according to the item intent descriptions provided by the HLQ developers, were analysed using thematic analysis. Information was collected on the comprehensibility of the questions, students’ understanding of questions and reasons for selecting their responses.

#### 2.5.3. The Internal Structure of the Measurement Instrument

Evidence based on the internal structure of the items and scales was examined through a series of nine single-factor confirmatory factor analysis (CFA) models to investigate scale homogeneity followed by a nine-factor CFA model to confirm that the hypothesized nine-scale structure of the HLQ was a good fit to the data and investigate, by exploring possible cross-loadings, whether each item was clearly associated with its hypothesized scale. The CFA models were also used to investigate if the data collected in Nepal can be compared with previous studies reporting factor structure for the HLQ [30,36,41] applied to this Nepalese population.

#### 2.5.4. Relations to Other Variables

The evidence for the relationship with the personal and sociodemographic characteristics of the students are published elsewhere in 2019 [15]. In this paper, we present an overview of the findings to the report as per ‘the Standards’. Cronbach’s alpha was calculated for each of the one-factor models.

#### 2.5.5. The Consequences of Testing

Discussions with university students and staff were used to investigate the consequences of measurement with the HLQ—that is, to understand if the results of the study reflected health literacy strengths and needs, as perceived by the health sciences students and staff, and if the resulting health literacy profiles indicated areas in which meaningful, implementable, and useful actions for health literacy development could be undertaken.

Discussions were held separately with students and teaching staff to examine the key findings of the study. The focus of the discussions was on the HLQ scales with higher and lower scores, as suggested by the participants. The discussions aimed to gather views about the relevance and implications of the results for the study population.

## 3. Results

Cognitive testing and cultural adaptation revealed that all items of the HLQ were understood well by the students and no changes were required.

A total of 419 students participated in the study. Over two-thirds of the participants (68.3%) were between 15 and 19 years and over half of the participants were male students (55.8%). About 62% of the participants had one parent who had completed university education. A huge proportion of students were enrolled in an undergraduate course, with 61% of the participants studying medicine. The socio-demographic characteristics of the study population along with the overall results of the study were published previously [15].

The Standards’ five sources of evidence framework was used to report the results of the validity testing process.

### 3.1. Evidence Based on the Content of the HLQ Items and Scales

The difficulty level for each HLQ item was assessed quantitatively for the survey respondents. For Part 1, the lowest average difficulty level was 17.4% for Scale *4*. *Social support for health* and the highest difficulty level was 30.4% for Scale *2*. *Having sufficient information to manage my health*. At the item level, the lowest difficulty level observed was 6.9% for the item *I feel I have good information about…* and the highest difficulty level was 41.3% for the item *I have all of the information I need…* Both these extremes were part of Scale 2. Having sufficient information to manage my health (Table 1).

For Part 2, the lowest average difficulty level was 39.9% for Scale *9*. *Understand health information well enough to know what to do* and the highest difficulty level was 51.9% for Scale *6. Ability to actively engage with healthcare providers*. At the item level, the lowest difficulty level observed was 34.1% for the item *Confidently fill in medical forms…* from Scale *9*. *Understand health information well enough to know what to do* and the highest difficulty level was 58.0% for the item *Make sure that healthcare providers…* from Scale *6*. *Ability to actively engage with health care providers* (Table 2).

### 3.2. Evidence Based on the Response Processes of HLQ Respondents

In-depth cognitive interviews were conducted with 10 students and 5 teaching staff in order to assess the extent to which respondents engaged with the English language items as intended by the HLQ developers. This was essential because the native language of the study population was not English, but rather the population uses English as an academic and professional language. No changes were deemed necessary.

### 3.3. Evidence Based on the Internal Structure of the HLQ

For the restricted nine-factor CFA model, the values indicate satisfactory level of fit (chi-square = 1993, df = 866, *p*-value < 0.0001, CFI = 0.945, TLI = 0.939 and RMSEA = 0.056)

Following the data analysis protocol outlined in Section 2.5, modification indices, in particular, the SEPC, were used to seek improvements to the highly restricted model, yielding a modified model with a chi-square = 1684.947, and *p*-value < 0.0001 was obtained. Other indices of goodness of fit obtained were CFI = 0.959, TLI = 0.955 and RMSEA = 0.048, indicating a good model fit. The factor loadings, R square and the model parameters for this model are shown in Table 1 and Table 2.

The modified model had four target loadings dropped (item: I know how to find out if the health information I receive… from Scale 5; items: Get to see the healthcare providers… and Work out what is the best… from Scale 7; and item: Get health information in… from Scale 8). There were four residual correlations and eight item cross-loadings in the final model. However, the cross-loading of the item I always compare health information… was dropped, and the remaining seven item cross-loadings of Scales 4, 6, 8 and 9 are presented separately in Table 3.

The one-factor CFA model showed good fit for three Scales: *2*. *Having sufficient information to manage my health* (CFI = 1, TLI = 1, RMSEA = 0), *7*. *Navigating the health care system* (CFI = 0.998, TLI = 0.996, RMSEA = 0.039) and *9*. *Understand health information well enough to know what to do* (CFI = 0.993, TLI = 0.985, RMSEA = 0.049). There was a reasonable fit seen for Scale *3*. *Actively managing my health* (CFI = 0.996, TLI = 0.992, RMSEA = 0.055) and Scale *4*. *Social support for health* (CFI = 0.992, TLI = 0.985, RMSEA = 0.071) (Table 1 and Table 2).

### 3.4. Evidence Based on the Relationships of the Scores to Other Variables

The results of the association of personal and sociodemographic characteristics of the study population are published in a 2019 paper [15]. In summary, male students had higher scores on Scales *2*. *Having sufficient information to manage my health*, *6*. *Ability to actively engage with healthcare providers*, and *8. Ability to find good health information* compared to female students. Older students (≥20 y of age) had higher for Scales *1*. *Feeling understood by my healthcare providers*, and *2*. *Having sufficient information to manage my health* than students who were less than 20 y of age. Participants whose parents had attained a university level of education had higher scores on Scale *7*. *Navigate the healthcare system* compared with students whose parents did not attain a university education. Postgraduate students scored higher on Scales *1*. *Feeling understood by my healthcare providers*, *2*. *Having sufficient information to manage my health* and *7*. *Able to navigate the healthcare system* compared to undergraduate students.

Cronbach’s alpha scores were of an acceptable or good level of reliability for most items (α = 0.6 or higher), except for three: *I have the healthcare providers I need to help me…* (α = 0.224) from Scale *1*, *Ask healthcare providers questions…* (α = 0.315) from Scale *6*, and *Find out what healthcare services you are…* (α = 0.275) from Scale *7*.

### 3.5. Evidence Based on Validity and the Consequences of Measurement Using the HLQ

For Part 1, the highest score was found for Scale 4. Social support for health and lowest score in Scale 1. Having sufficient information to manage their health. For Part 2, the highest score was found for Scale 9. Understanding health information well enough to know what to do and the lowest score on Scale 6. Ability to actively engage with healthcare providers. The discussions in both the student and teaching staff groups separately came to the agreement that the overall results are convincing and realistically reflect their individually perceived health literacy strengths and needs of the population. As suggested by the participants, the potential health literacy development initiative to support students to access health information was to digitize the central library resources. For improving active engagement with healthcare providers, the participants suggested conducting workshops to engage students with the healthcare providers, who are usually their teachers and clinical supervisors.

## 4. Discussion

This validity testing paper reports the first-ever use of HLQ in a Nepali population. This study is unique in another way in that it reports the use of the English HLQ in a Nepali speaking population who use English as their academic and professional language. Additionally, this paper presents the study methods and results according to the five sources of validity evidence from the Standards for Educational and Psychological Testing [48], which gives a clear framework in which to describe what the data mean for score interpretation and use and enable the evidence to tell a story about the HLQ in the Nepali context.

The five sources of evidence are best employed prospectively to gather information about the interpretation and use of data generated by a new questionnaire or of data generated by a questionnaire in a new context [55]. This study used a questionnaire in a new context. The HLQ is a questionnaire that is well established in the field of health literacy measurement [56]. Having a tool that generates valid multidimensional health literacy data is an opportunity to promote the assessment of health literacy strengths, needs and preferences of patients and communities. However, the HLQ had not previously been used in Nepal, in English, with a largely Nepali-speaking population. The study population was health sciences students who will graduate to work as clinicians and healthcare professionals in Nepal and around the world, who are likely to mostly use English as their professional language. The validity of the interpretation and use of HLQ data in this context was unknown. Investigations were required into all aspects of the questionnaire, including potential consequences of using the data for making decisions about initiatives to develop the health literacy of the health sciences students who are the future healthcare professionals. The use of HLQ has shown that Nepalese health sciences students have good information about health but may not have adequate skills to take care of their health. This could be because they have limited skills for using health knowledge to make healthcare decisions. They also express difficulty in engaging in discussion with their healthcare providers who are their teachers in medical school [15]. This could be at least partly explained to be an influence of the existing social hierarchy that places doctors, teachers and professionals at a higher social level in Nepali culture than students.

As a collectivistic society, the sense of social support would generally be expected to be higher than reported in [15]. This opens up opportunities for future studies to consider the role of interprofessional education to support improved health literacy of health science students because Nepalese health sciences education also incorporates the multidisciplinary education of medical, dental and nursing students in the curriculum [57].

Given the very restricted model, its fit is comparable to the fit of other psychometric analyses of the English-language HLQ [30]. The satisfactory model fit seen in this paper may not only apply to the health sciences students and could possibly be generalizable to healthcare professionals who use English as an academic and professional language. However, given the large number of parameters (236) estimated in our sample of 419 observations, we acknowledge that out-of-sample performance needs further investigation.

When measurements are undertaken, it implies some action will take place [58], and these actions need to be appropriate, meaningful, and useful for the target population. Health professionals must understand and address their own health literacy strengths, needs and preferences because this may enable professionals to more quickly recognize their patients’ health literacy strengths, needs and preferences and to engage their patients in meaningful discussions to co-design appropriate health actions or interventions.

Given that the HLQ was developed in a Western context, it is important to establish that the content of the HLQ’s items is meaningful in the Nepalese context. The synthesis of the investigations in this study reveals that the content and meaning of the English HLQ items are appropriate for the study population (content and response processes investigations), that the items and scales function in the same way within each scale and between scales in this population as they do in the population in the HLQ development study (investigations into internal structure and relations to other variables) [30], and that the results of the study resonate with and are meaningful to the members of the study population, which indicates that the data are likely to be useful for informing health literacy development initiatives to support health sciences students in Nepal now and into their futures.

With the increasing incidence of NCDs around the world, the responsibility of creating medical environments that support the prevention and control of these conditions will fall to the health professionals of the future, as well as clinical researchers, managers and policymakers [59]. Harnessing the health literacy strengths and understanding and responding to the health literacy needs of diverse populations will be essential components of healthcare delivery [1]. Early training in health literacy will be an asset to all students of health sciences and medical disciplines, as will be understanding their own health literacy strengths and needs [60].

## 5. Conclusions

The health literacy development implications of the HLQ validity testing in this study is twofold: first is the potential for using the English HLQ among health professionals, not only students, in Nepal; and second is the potential for using the English HLQ among health professionals from non-English-speaking countries who work in English-speaking countries around the world. The analyses suggest that the nine HLQ constructs appear to be potentially meaningful for use in Nepal. However, having HLQ translated into Nepali and a few dominant languages for specific community populations within Nepal is a useful next step. Given the societal and cultural differences between high-income English-speaking countries, where the communities are more individualistic, and Nepal where the community is more collective, careful consideration of the cultural appropriateness through cognitive interviews is still essential.

## Figures and Tables

**Table 1:** Difficulty level and psychometric properties of English HLQ among students in Nepal (Part 1 Scales *1*–*5*).

Scale/Item	n	Difficulty Level (%)	Average Item Difficulty (%)	One-Factor CFA			Nine-Factor CFA (Unrestricted)	
				Factor Loadings	R^2^	Cronbach’s Alpha	Factor Loadings	R^2^
Part 1: Scales *1*–*5*: How strongly you disagree or agree with the following statements (strongly disagree/disagree/disagree/agree/strongly agree)								
1. Feeling understood and supported by healthcare providers								
I have at least one healthcare provider…	419	20.1	21.5	0.750	0.562	0.797	0.713	0.508
I have at least one healthcare provider …	419	24.8		0.851	0.724		0.799	0.638
I have the healthcare providers I need to help me …	419	18.6		0.709	0.503		0.224	0.658
I can rely on at least one…	419	22.4		0.790	0.624		0.889	0.791
*Model fit (one-factor CFA) chi square = 55.839, p-value = 0, CFI = 0.971, TLI = 0.913, RMSEA = 0.253, SRMR = 0.033*								
2. Having sufficient information to manage my health								
I feel I have good information about…	419	6.9	30.2	0.581	0.337	0.794	0.647	0.418
I have enough information to help me…	419	32.0		0.817	0.668		0.879	0.772
I am sure I have all the information I need…	419	40.8		0.838	0.702		0.823	0.677
I have all of the information I need…	419	41.3		0.886	0.785		0.829	0.687
*Model fit (one-factor CFA) chi square = 1.924, p-value = 0.382, CFI = 1, TLI = 1, RMSEA = 0, SRMR = 0.007*								
3. Actively managing my health								
I spend quite a lot of time actively…	419	33.4	22.4	0.699	0.488	0.786	0.639	0.408
I make plans for what I need to do…	419	22.4		0.810	0.657		0.792	0.627
Despite other things in my life, I make time to…	419	17.9		0.781	0.610		0.714	0.509
I set my own goals…	419	18.6		0.654	0.427		0.740	0.547
There are things that I do regularly…	419	19.8		0.735	0.540		0.773	0.598
*Model fit (one-factor CFA) chi square = 11.446, p-value = 0.043, CFI = 0.996, TLI = 0.992, RMSEA = 0.055, SRMR = 0.017*								
4. Social support for health								
I can get access to several people…	419	12.7	17.4	0.764	0.584	0.767	0.730	0.532
When I feel ill, the people…	419	25.3		0.541	0.292		0.613	0.375
If I need help, I have plenty of…	419	21.5		0.797	0.635		0.804	0.646
I have at least one person who can come to…	419	19.1		0.676	0.457		0.612	0.375
I have strong support from family…	419	8.6		0.749	0.561		0.677	0.459
*Model fit (one-factor CFA) chi square = 15.692, p-value = 0.008, CFI = 0.992, TLI = 0.985, RMSEA = 0.071, SRMR = 0.02*								
5. Appraisal of health information								
I compare health information…	419	18.4	22.6	0.681	0.464	0.738	0.622	0.387
When I see new information about health…	419	27.9		0.710	0.504		0.622	0.386
I always compare health information…	419	21.0		0.730	0.532		0.650	0.422
I know how to find out if the health information I receive…*	419	19.6		0.605	0.366		-	-
I ask healthcare providers about…	419	26.0		0.629	0.396		0.877	0.769
*Model fit (one-factor CFA) chi square = 43.957, p-value = 0, CFI = 0.962, TLI = 0.923, RMSEA = 0.136, SRMR = 0.038*								

* target loadings dropped in unrestricted nine-factor CFA.

**Table 2:** Difficulty level and psychometric properties of English HLQ among students in Nepal (Part 2, Scales *6*–*9*).

Scale/Item	n	Difficulty Level (%)	Average Item Difficulty (%)	One-Factor CFA			Nine-Factor CFA (Unrestricted)	
				Factor Loadings	R^2^	Standardized Cronbach’s Alpha	Factor Loadings	R^2^
Part 2: Scales *6*–*9*: How easy or difficult the following tasks are for you to do now (cannot do or always difficult/usually difficult/sometimes difficult/usually easy/always easy)								
6. Ability to actively engage with health care providers								
Make sure that healthcare providers…	419	58.0	51.9	0.748	0.560	0.881	0.762	0.580
Feel able to discuss your health…	419	48.9		0.840	0.705		0.824	0.680
Have good discussions about…	419	48.9		0.856	0.733		0.906	0.649
Discuss things with healthcare…	419	52.3		0.847	0.718		0.866	0.750
Ask healthcare providers questions…	419	51.6		0.815	0.664		0.315	0.698
*Model fit (one-factor CFA) chi square = 36.357, p-value = 0, CFI = 0.992, TLI = 0.983, RMSEA = 0.122, SRMR = 0.018*								
7. Navigating the health care system								
Find the right…	419	52.0	49.6	0.589	0.347	0.841	0.606	0.368
Get to see the healthcare providers you… *	419	50.8		0.695	0.484		-	-
Decide which healthcare provider…	419	49.9		0.824	0.680		0.799	0.639
Make sure you find the right place…	419	45.1		0.821	0.675		0.865	0.748
Find out what healthcare services you are…	419	43.0		0.784	0.614		0.275	0.659
Work out what is the best… *	419	56.6		0.713	0.508		-	-
*Model fit (one-factor CFA) chi square = 14.696, p-value = 0.099, CFI = 0.998, TLI = 0.996, RMSEA = 0.039, SRMR = 0.015*								
8. Ability to find good health information								
Find information about…	419	47.0	50.0	0.674	0.455	0.839	0.690	0.477
Find health information from several different…	419	55.4		0.798	0.637		0.783	0.612
Get information about health so you …	419	57.3		0.804	0.646		0.798	0.637
Get health information in…*	419	38.0		0.765	0.586		-	-
Get health information…	419	52.3		0.795	0.632		0.820	0.672
*Model fit (one-factor CFA) chi square = 32.792, p-value = 0, CFI = 0.988, TLI = 0.976, RMSEA = 0.115, SRMR = 0.023*								
9. Understand health information well enough to know what to do								
Confidently fill in medical forms…	419	34.1	39.9	0.662	0.438	0.823	0.738	0.545
Accurately follow instructions…	419	50.6		0.600	0.360		0.685	0.469
Read and understand…	419	35.1		0.732	0.536		0.784	0.614
Read and understand all the…	419	39.6		0.738	0.545		0.744	0.554
Understand what healthcare providers…	419	40.1		0.738	0.545		0.824	0.679
*Model fit (one-factor CFA) chi square = 9.986, p-value = 0.0756, CFI = 0.993, TLI = 0.985, RMSEA = 0.049, SRMR = 0.018*								
*Model fit (nine-factor CFA) chi square = 1684.947, p-value = 0.0000, CFI = 0.959, TLI = 0.955, RMSEA = 0.048, SRMR = 0.049*								

* target loadings dropped in unrestricted nine-factor CFA.

**Table 3:** Cross-loadings of items in different factors obtained through unrestricted nine-factor CFA.

Scale/Item	n	Nine-Factor CFA (Unrestricted)	
		Factor Loadings	R^2^
4. Social support for health			
I know how to find out if the health information I receive is…	419	0.846	0.715
I have the healthcare providers I need to help me…	419	0.63	0.658
6. Ability to actively engage with healthcare providers			
Get to see the healthcare providers you…	419	0.779	0.608
8. Ability to find good health information			
Work out what is the best…	419	0.742	0.551
9. Understand health information well enough to know what to do			
Get health information in…	419	0.852	0.726
Ask healthcare providers questions…	419	0.547	0.698
Find out what healthcare services you are…	419	0.561	0.659

## Data Availability

The data presented in this study are available on request from the corresponding author.
